# Changes in vitamin and mineral supplement use after breast cancer diagnosis in the Pathways Study: a prospective cohort study

**DOI:** 10.1186/1471-2407-14-382

**Published:** 2014-05-29

**Authors:** Heather Greenlee, Marilyn L Kwan, Isaac J Ergas, Garrett Strizich, Janise M Roh, Allegra T Wilson, Marion Lee, Karen J Sherman, Christine B Ambrosone, Dawn L Hershman, Alfred I Neugut, Lawrence H Kushi

**Affiliations:** 1 Mailman School of Public Health, Columbia University, New York, NY 10032, USA; 2 Herbert Irving Comprehensive Cancer Center, Columbia University Medical Center, New York, NY 10032, USA; 3 Division of Research, Kaiser Permanente Northern California, Oakland, CA 94612, USA; 4 University of California San Francisco, San Francisco, CA 94143, USA; 5 Group Health Research Institute, Seattle, WA 98101, USA; 6 Roswell Park Cancer Institute, Buffalo, NY 14263, USA; 7 College of Physicians and Surgeons, Columbia University, New York, NY 10032, USA

**Keywords:** Breast cancer, Cohort studies, Vitamins, Multivitamins, Dietary supplements

## Abstract

**Background:**

Vitamin and mineral supplement use after a breast cancer diagnosis is common and controversial. Dosages used and the timing of initiation and/or discontinuation of supplements have not been clearly described.

**Methods:**

We prospectively examined changes in use of 17 vitamin/mineral supplements in the first six months following breast cancer diagnosis among 2,596 members (28% non-white) of Kaiser Permanente Northern California. We used multivariable logistic regression to examine demographic, clinical, and lifestyle predictors of initiation and discontinuation.

**Results:**

Most women used vitamin/mineral supplements before (84%) and after (82%) diagnosis, with average doses far in excess of Institute of Medicine reference intakes. Over half (60.2%) reported initiating a vitamin/mineral following diagnosis, 46.3% discontinuing a vitamin/mineral, 65.6% using a vitamin/mineral continuously, and only 7.2% not using any vitamin/mineral supplement before or after diagnosis. The most commonly initiated supplements were calcium (38.2%), vitamin D (32.01%), vitamin B6 (12.3%) and magnesium (11.31%); the most commonly discontinued supplements were multivitamins (17.14%), vitamin C (15.97%) and vitamin E (45.62%). Higher education, higher intake of fruits/vegetables, and receipt of chemotherapy were associated with initiation (p-values <0.05). Younger age and breast-conserving surgery were associated with discontinuation (p-values <0.05).

**Conclusions:**

In this large cohort of ethnically diverse breast cancer patients, high numbers of women used vitamin/mineral supplements in the 6 months following breast cancer diagnosis, often at high doses and in combination with other supplements. The immediate period after diagnosis is a critical time for clinicians to counsel women on supplement use.

## Background

American women, especially those diagnosed with cancer, commonly use dietary supplements [1,2]. Recent studies have reported that 36-87% of U.S. breast cancer survivors regularly take a form of vitamin or mineral supplement following diagnosis [3-12], with use more common among women who are older [12] or have more education [8]. Common reasons for use include the belief that it will increase tolerance to conventional treatment, alleviate treatment side effects, manage hot flashes, boost immune function, and promote general health [13].

The efficacy of supplement use for improvement of health outcomes following a breast cancer diagnosis remains unclear. The majority of studies have not found dietary supplements to improve breast cancer prognosis [5,14,15], with some showing benefit [4,6,16] and some showing harm [4]. Controversy exists regarding use of antioxidant supplements during treatment due to potential interactions with conventional therapy [17,18] and lack of clear evidence-based clinical guidelines. A recent study showed that patient and physician discussions about supplement use following a breast cancer diagnosis is uncommon [19].

Many prior studies examining the association between supplement use pre- and post-cancer diagnosis with cancer-related outcomes have had methodological limitations. These limitations included lack of detailed prospective data on supplement use, specifically around the time of diagnosis and treatment, as well as lack of data collection on changes in supplement use over time. In addition, supplement use prior to diagnosis is often not assessed and may confound associations between later supplement use and cancer outcomes [6].

We examined change in use of vitamin and mineral supplements within the first six months following breast cancer diagnosis in a prospective cohort of breast cancer survivors in Kaiser Permanente Northern California.

## Methods

### Participants

The Pathways Study is a prospective study of women with newly diagnosed invasive breast cancer who are members of Kaiser Permanente Northern California (KPNC), a large, integrated health care delivery system covering the San Francisco-Oakland Bay Area, Sacramento, and surrounding counties. Recruitment was from January 2006 to April 2013 through rapid case ascertainment procedures designed to enroll women prior to initiation of chemotherapy, as described elsewhere [20]. Briefly, cases were ascertained through daily scanning of computerized pathology records for any new patients with recently diagnosed breast cancer (usually within 2 months). Participation was restricted to KPNC female members at least 21 years of age; had no previous history of malignancy other than non-melanoma skin cancer; spoke English, Spanish, Cantonese, or Mandarin; and resided within a 65-mile radius of a field interviewer. The study was approved by the Columbia University Institutional Review Board; the Kaiser Permanente Northern California Institutional Review Board; the University of California, San Francisco Committee on Human Research; and the Roswell Park Cancer Institute Institutional Review Board.

Breast cancer diagnosis and patient notification of diagnosis were verified through manual review of electronic records, and passive physician consent was obtained prior to participant recruitment. Written informed consent was obtained from all participants at the baseline interview. Participation rates and reasons for refusal have been described previously [20]. Potentially eligible women (n = 11,233) were invited to participate in the study, and 4,505 enrolled (approximately 47%) after taking into account number of ineligibles, no contacts, and refusals). Primary reasons for refusal include being uninterested, too busy, too tired, other health issues, feeling overwhelmed, and/or participation in another study. Analyses presented here include 2,596 women who completed both the baseline and six-month follow-up questionnaires as of February 19, 2014, had a record in the KPNC Cancer Registry as of June December 31, 2012, and specified their race/ethnicity. The mean time from diagnosis to enrollment was 2.0 (±0.7) months, and from enrollment to follow-up 6.0 (±1.0) months.

### Measures

Clinical and diagnostic tumor characteristics were obtained from the KPNC Cancer Registry approximately four months post-diagnosis. These included: AJCC stage at diagnosis, number of positive nodes, estrogen/progesterone receptor positivity, HER2/neu status, surgery type, and treatments received. The baseline interview was conducted at enrollment approximately two months post-diagnosis and included interviewer and self-administered questionnaires on sociodemographics, diet, physical activity, smoking, established breast cancer risk factors, health history, and use of vitamin/mineral supplements. Anthropometric measures were also obtained at baseline. Six months after the baseline interview, a packet of follow-up materials soliciting the same information collected at baseline was mailed to the participant’s home, with interviewer assistance offered if needed.

### Assessment of change in vitamin/mineral supplement intake

At baseline and the six month follow-up, each participant was asked about multivitamins and single formulations taken at least once a week for a month or longer, with container labels being referenced if available. Women were first asked whether they had ever taken each type of supplement. For those reporting ever use of a particular supplement, consumption prior to breast cancer diagnosis was assessed for each single supplement by asking, “Did you take (vitamin/mineral) before you were diagnosed?” and for multivitamins by asking, “How many times per week did you take the multivitamin before your diagnosis?” Detailed information was then obtained regarding dose, frequency and duration of use. Multivitamin dose was assessed using the number of pills taken each time for multivitamins. Single supplement dose categories were based on common doses used in store-bought formulations.

Baseline and follow-up questionnaire responses were used to establish supplement use during the two time periods of interest: 1) prior to diagnosis and 2) between baseline and the six-month follow-up interview. Use after diagnosis was defined as any yes response during the period between the baseline and six-month follow-up interviews. These data were used to identify four classifications of users for each supplement: initiators, discontinuers, continuous users, and non-users. Initiators were those using supplements after baseline but not prior to diagnosis. Discontinuers were defined as those reporting use before diagnosis but not at any time between baseline and the follow-up interview. Continuous users were those using the specified supplement both before diagnosis and at any point between baseline and follow-up. Non-users reported no use prior to or following diagnosis.

### Baseline assessment of diet and physical activity

Diet was assessed using a 139-item modified version of the Block 2005 food frequency questionnaire (NutritionQuest, Berkeley, CA). Food items were selected by identifying the top population contributors of each nutrient among whites, African Americans, and Hispanics in the National Health and Nutrition Examination Survey (1999-2002) [21,22]. Physical activity was assessed with an activity frequency questionnaire based on the Arizona Activity Frequency Questionnaire [23].

### Statistical analyses

Bivariate analyses of ever use of vitamin/mineral supplements, stratified by race/ethnicity, were performed using Pearson’s chi-squared test. Next, the frequencies of never use, continuous use, initiating, and discontinuing were calculated for each supplement. To better understand patterns of use in continuous users, mean doses were established for each supplement, and changes in dose were computed by subtracting the average daily dose prior to diagnosis from the average daily dose reported at the six-month follow-up. Compared to baseline, a positive or negative value indicates an increase or decrease in dose at follow-up, respectively. Student’s t-tests were used to examine differences in dose between continuous users and initiators or discontinuers. Doses for initiators were compared with doses for continuous users at the six-month follow-up, whereas doses for discontinuers were compared with doses of continuous users prior to diagnosis.

Multivariable logistic regression was performed to explore predictors of initiation, discontinuation, and continuous use of multivitamins, vitamin C, vitamin D, vitamin E and calcium, which were the most commonly used. Models were adjusted for age, race, education, household income, family history of breast cancer, stage at diagnosis, number of positive lymph nodes, hormone receptor positivity, HER2/neu status, surgery type, treatment received, BMI, fruit/vegetable intake, physical activity and smoking status. Prior use of vitamins/minerals was not included as a covariate in the models due to the possibility of spurious associations between predictors of interest and change in supplement use. Odds ratios (OR) and 95% confidence intervals (CI) for initiation were calculated among non-users prior to diagnosis. Similarly, among supplement users prior to diagnosis, the odds of discontinuing by the six-month follow-up questionnaire were calculated. All p-values were two-tailed with a significance level of 0.05.

## Results

### Participant characteristics

Demographic and clinical characteristics for women completing both the baseline and six-month questionnaires are presented in Table 1. Study participants represent an ethnically diverse population: 71.5% non-Hispanic whites, 11.9% Asians, 10.6% Hispanics, and 6.0% African Americans. The median age at diagnosis was 61.1 ± 11.8 years, and the average education level was college graduate. Most participants had either stage I (56.2%) or stage II (33.0%) breast cancer at diagnosis, and the majority had tumors positive for both estrogen and progesterone receptors (64.1%).

**Table 1:** **Baseline demographic characteristics, clinical characteristics and use of vitamin/mineral supplements prior to diagnosis, by race, Kaiser Permanente Northern California, 2006-2013 (n = 2,596**^**a**^**)**

	**All (n = 2,596) %**	**Non-Hispanic White (n = 1,857) %**	**African American (n = 155) %**	**Asian (n = 309) %**	**Hispanic (n = 275) %**	**P-value**^**b**^
**Demographic characteristics**						
Age						<.0001
<50	18.5	13.0	19.4	39.5	31.3	
50-59	26.7	24.8	35.5	30.4	29.8	
60-69	31.9	35.2	27.7	22.7	22.9	
70+	23.0	27.0	17.4	7.4	16.0	
Education						<.0001
High school or less	13.6	11.8	19.4	8.1	28.4	
Some college	33.4	35.1	42.6	14.9	37.8	
College graduate	28.4	26.7	22.6	49.5	19.6	
Post graduate	24.5	26.3	15.5	27.5	14.2	
**Clinical characteristics**						
Stage at diagnosis						0.13
I	56.2	57.3	50.3	54.7	54.2	
II	33.0	32.2	37.4	35.0	34.2	
III	9.6	9.4	9.0	10.0	11.3	
IV	1.1	1.2	3.2	0.32	0.36	
Hormone-receptor positivity						<.0001
ER-/PR-	15.7	13.9	29.0	17.2	18.2	
ER-/PR+	0.12	0.05	0.0	0.0	0.73	
ER+/PR-	20.0	21.1	22.6	15.5	16.0	
ER+/PR+	64.1	64.8	47.7	67.3	65.1	
Unknown	0.15	0.16	0.65	0.0	0.0	
**Use of vitamin/mineral supplements prior to diagnosis**						
Any vitamin/mineral	83.6	85.9	82.6	78.3	73.8	<.0001
Multivitamins	70.0	73.0	66.5	64.7	58.2	<.0001
Vitamin C	32.3	34.7	27.7	26.5	24.7	0.0003
Calcium, tums, or antacids with calcium	23.6	25.1	20.6	21.4	17.8	0.03
Vitamin E	21.9	23.4	22.6	17.2	16.7	0.01
Vitamin D	15.4	16.0	20.0	10.4	14.5	0.02
Iron	12.2	11.5	24.5	10.4	12.4	<.0001
Vitamin B12	9.6	9.4	16.1	6.5	11.3	0.007
Folic acid (folate)	8.4	8.9	9.7	6.5	6.9	0.36
Magnesium	5.2	5.7	5.8	1.6	5.5	0.03
Zinc	4.7	5.2	3.2	2.9	4.0	0.24
Vitamin A	3.2	3.1	4.5	1.6	4.7	0.13
Chromium	2.7	2.7	2.6	1.9	4.0	0.49
Niacin (B3)	2.0	2.2	1.9	1.0	1.8	0.58
Beta carotene	1.7	2.0	0.6	0.6	1.1	0.19
Selenium	1.8	2.2	1.9	0.3	1.5	0.16
Thiamin (B1)	0.8	0.9	0.0	0.6	1.1	0.64
**Use of vitamin/mineral supplements after diagnosis**						
Any vitamin/mineral	82.0	84.7	79.4	77.3	71.3	<.0001
Multivitamins	60.8	63.6	51.6	57.6	50.5	<.0001
Vitamin C	24.7	26.5	18.1	22.3	18.9	0.003
Calcium, tums, or antacids with calcium	51.8	56.2	38.7	44.0	38.5	<.0001
Vitamin E	11.6	12.8	7.7	7.8	10.5	0.02
Vitamin D	43.1	45.4	40	36.2	37.5	0.003
Iron	7.5	6.9	14.8	7.4	7.0	0.009
Vitamin B12	13.8	14.6	16.1	10.4	11.3	0.11
Folic acid (folate)	9.1	10.1	7.1	5.8	7.3	0.04
Magnesium	13.8	15.6	7.1	10.0	10.2	0.0004
Zinc	7.6	8.6	5.2	6.5	3.6	0.01
Vitamin A	3.1	3.2	1.9	2.6	3.3	0.75
Chromium	3.0	3.2	1.9	2.3	2.9	0.66
Niacin (B3)	7.4	8.3	5.2	4.5	5.5	0.03
Beta carotene	1.7	1.9	0.65	1.6	1.5	0.67
Selenium	3.1	3.5	1.9	1.9	2.2	0.27
Thiamin (B1)	6.5	7.1	3.9	5.5	5.8	0.31

^a^Among Pathways participants with both baseline and 6 month follow up questionnaire.

^b^Calculated using a chi-squared test.

### Overall vitamin/mineral supplement use

Most women (83.6%) reported using at least one vitamin/mineral supplement prior to diagnosis, with the most common being multivitamins (70.0%) (Table 1). Prior to diagnosis, vitamin C, calcium, and vitamin E were each used by over 20% of women, and vitamin D was used by 15.4% of women. Vitamin/mineral supplement use prior to diagnosis was highest among non-Hispanic whites (85.9%) and lowest among Hispanics (73.8%).

### Changes in vitamin/mineral supplement use

**Figure 1:**
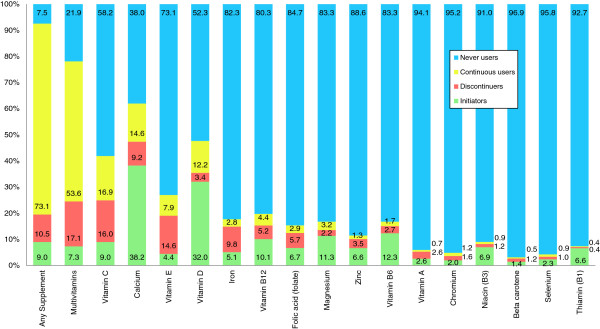
**Change in supplement use from breast cancer diagnosis to 6 months post diagnosis.** For each type of dietary supplement, this figure displays the percent of participants who were never users (blue), continuous users (yellow), discontinuers (red) and initiators (green) from the time of breast cancer diagnosis to 6 months post diagnosis.

At the six-month follow-up, 82.0% of the study population reported using at least one vitamin/mineral supplement following diagnosis with breast cancer (Table 1). Over half (60.2%) of women reported initiating a vitamin/mineral following diagnosis, 46.3% reported discontinuing a vitamin/mineral, 65.5% reported using a vitamin/mineral continuously, and only 7.2% reported not using any vitamin/mineral supplement before or after diagnosis (data not shown). Figure 1 shows the number of initiators, discontinuers, continuous users and never users for each type of vitamin/mineral supplement. Calcium (38.2%), vitamin D (32.0%), magnesium (11.3%), and vitamin B6 (10.1%) were the most commonly initiated supplements. Multivitamins (17.1%), vitamin C (16.0%), vitamin E (14.6%), calcium (9.2%), iron (9.8%), and folic acid (5.7%) were the most commonly discontinued supplements. Multivitamins (53.6%), vitamin C (16.9%), calcium (14.6%), and vitamin D (12.2%) were the most commonly used continuously from the period before diagnosis through the 6-month follow-up.

### Doses of vitamin/mineral supplements

Doses of nearly all vitamin/mineral supplements were far in excess of the Institute of Medicine’s (IOM) Dietary Reference Intakes [24] (Table 2). Among continuous users, there were notable increases in the mean consumption of vitamin D (33.8%), magnesium (18.1%) and calcium (17.0%), and substantial decreases in vitamin A (31.9%) and beta-carotene (21.1%). On average, continuous users consumed higher doses of supplements than initiators or discontinuers for most supplements, although doses were often still in excess of recommended levels.

**Table 2:** **Change in vitamin/mineral supplement daily dose from diagnosis to 6 months**^**a**^

		**Initiators**^**b**^		**Discontinuers**^**c**^		**Continuous users**		
	**IOM reference intakes**^**d**^	**Change in dose from diagnosis to 6 months**	**P-value**^**e**^	**Change in dose from diagnosis to 6 months**	**P-value**^**f**^	**Dose at diagnosis**	**Dose at 6 months**	**Change from diagnosis**^**g**^
Multivitamins (pills)	n/a	1.45	0.53	−1.32	0.34	1.39	1.23	−0.17
Vitamin C (mg)	75 mg	615.95	0.01	−557.97	<.0001	780.10	740.57	−26.07
Calcium (mg)	1200 mg	859.31	<.0001	−743.69	0.56	704.55	815.69	119.70
Vitamin E (IU)	15 mcg^h^	280.89	<.0001	−391.09	0.66	400.97	357.97	−41.32
Vitamin D (IU)	600 mcg^g^	1064.94	<.0001	−2505.33	0.26	1800.20	2306.79	609.09
Iron (mg)	8 mg	184.13	0.56	−80.08	0.38	129.99	79.66	−73.85
Vitamin B12 (mcg)	2.4 mcg	245.80	<.0001	−612.35	0.18	794.29	625.99	−126.84
Folic acid (folate) (mcg)	400 mcg	416.38	<.0001	−475.81	0.05	616.75	552.73	−42.47
Magnesium (mg)	320 mg	289.02	0.91	−315.55	0.62	293.46	319.25	57.69
Zinc (mg)	8 mg	12.79	<.0001	−17.56	0.004	42.14	38.08	4.46
Vitamin B6 (mg)	1.3-1.5 mg	63.77	0.07	−110.61	0.56	88.96	91.34	−16.14
Vitamin A (IU)	700 mcg^i,h^	5966.11	0.02	−11113.52	0.93	10736.26	7283.16	−3425.60
Chromium (mg)	0.02 mg	165.46	0.03	−176.16	0.09	280.96	284.16	13.65
Niacin (B3) (mg)	14 mg	167.02	<.0001	−368.21	0.02	850.00	915.86	94.52
Beta carotene (IU)		12552.93	0.10	−12750.00	0.15	19722.22	13437.50	−4166.67
Selenium (mcg)	55 mg	101.96	0.11	−111.01	0.21	144.25	137.41	−1.28
Thiamin (B1) (mg)	1.1 mg	57.81	0.07	−28.35	0.17	164.06	179.76	57.14

^a^Ordered by prevalence of use prior to diagnosis.

^b^Initiators defined as those taking supplements at six-month follow-up but not at baseline visit.

^c^Discontinuers defined as those taking supplements at baseline but not at six-month follow-up visit.

^d^Based on Institute of Medicine Recommended Dietary Allowances and Adequate Intakes for females age 51 years and older.

^e^T-test comparing 6 months dose to baseline dose of Continuous Users.

^f^T-test comparing diagnosis dose to diagnosis dose of Continuous Users.

^g^The amount of change may not reflect the apparent difference between diagnosis and 6 months since both measures are required in order to be included.

^h^IOM recommended dietary allowances for vitamins A, D, and E are expressed in micrograms, while the study values are international units (I.U.).

^i^The IOM recommended dietary allowance of 700 mcg vitamin A is expressed in retinol activity equivalents (RAE), where 1 RAE = 1 mcg retinol or 12 mcg beta-carotene.

### Predictors of supplement initiation, discontinuation and continuous use

Tables 3, 4 and 5 show ORs and 95% CIs from multivariable-adjusted models for initiation, discontinuation and continuous use of the five most commonly used supplements after breast cancer diagnosis, among women who were and were not using the specific supplement prior to diagnosis, respectively.

**Table 3:** Predictors of initiating vitamin/mineral supplement use by the 6-month follow up

**Covariates**	**Multivitamins (n = 751)**			**Vitamin C (n = 1,661)**			**Calcium (n = 1,941)**			**Vitamin E (n = 1,897)**			**Vitamin D (n = 2,129)**		
	**OR**	**95% CI**	**P-value**	**OR**	**95% CI**	**P-value**	**OR**	**95% CI**	**P-value**	**OR**	**95% CI**	**P-value**	**OR**	**95% CI**	**P-value**
**Age**															
<50 years	Ref			Ref			Ref			Ref			Ref		
50-59 years	1.03	0.59, 1.81	0.97	0.78	0.48, 1.25	0.30	2.56	1.84, 3.55	<.0001	0.68	0.37, 1.26	0.22	1.82	1.34, 2.48	.0001
60-69 years	0.62	0.34, 1.14	0.13	0.92	0.56, 1.49	0.72	3.01	2.13, 4.23	<.0001	0.42	0.22, 0.83	0.01	2.19	1.59, 3.02	<.0001
70+ years	0.68	0.32, 1.42	0.30	0.98	0.56, 1.74	0.95	3.08	2.06, 4.60	<.0001	0.45	0.21, 0.97	0.04	2.02	1.38, 2.95	.0003
**Race**															
Non-Hispanic white	Ref			Ref			Ref			Ref			Ref		
African American	0.69	0.30, 1.30	0.39	0.76	0.36, 1.61	0.47	0.72	0.45, 1.16	0.17	0.75	0.28, 2.01	0.56	0.89	0.55, 1.43	0.63
Asian	0.91	0.47, 1.75	0.77	1.61	0.99, 2.62	0.05	0.83	0.58, 1.19	0.31	1.07	0.52, 2.21	0.85	0.87	0.62, 1.23	0.43
Hispanic	0.85	0.46, 1.56	0.60	0.77	0.38, 1.26	0.23	0.58	0.40, 0.83	0.004	1.20	0.60, 2.41	0.61	0.96	0.68, 1.37	0.83
**Education**															
Highschool or less	Ref			Ref			Ref			Ref			Ref		
Some college	2.58	1.35, 4.94	0.004	1.09	0.63, 1.91	0.75	0.91	0.64, 1.29	0.59	1.51	0.66, 3.45	0.33	0.84	0.58, 1.13	0.22
College graduate	1.61	0.78, 3.29	0.20	1.62	0.92, 2.86	0.10	1.14	0.79, 1.65	0.49	2.06	0.88, 4.83	0.10	1.00	0.70, 1.41	0.98
Post graduate	2.80	1.35, 5.78	0.006	0.99	0.53, 1.83	0.62	1.23	0.84, 1.81	0.30	2.11	0.87, 5.12	0.10	1.11	0.77, 1.60	0.59
**Household income**															
≤$25,000	Ref			Ref			Ref			Ref			Ref		
$25,000-49,999	0.43	0.23, 0.82	0.01	1.12	0.59, 2.13	0.73	1.21	0.81, 1.82	0.35	0.93	0.40, 2.17	0.87	1.18	0.79, 1.75	0.42
$50,000-89,999	0.25	0.13, 0.49	<.0001	1.28	0.69, 2.36	0.44	1.37	0.92, 2.03	0.12	1.04	0.46, 2.33	0.93	1.35	0.91, 2.0	0.13
$90,000+	0.29	0.15, 0.56	.0003	1.25	0.66, 2.36	0.49	1.49	0.99, 2.26	0.06	0.53	0.22, 1.28	0.16	1.22	0.81, 1.82	0.34
**Family history of breast cancer**															
No	Ref			Ref			Ref			Ref			Ref		
Yes	0.97	0.60, 1.57	0.91	1.17	0.80, 1.70	0.43	0.98	0.75, 1.26	0.86	1.54	0.95, 2.50	0.08	0.97	0.76, 1.24	0.80
**Stage at diagnosis**															
I	Ref			Ref			Ref			Ref			Ref		
II	1.0	0.54, 1.85	1.0	0.77	0.46, 1.28	0.31	0.71	0.51, 0.97	0.03	0.94	0.48, 1.84	0.86	0.88	0.65, 1.19	0.40
III+	0.50	0.12, 2.08	0.34	1.33	0.46, 3.82	0.59	0.49	0.23, 1.05	0.07	2.09	0.53, 8.30	0.30	0.65	0.31, 1.38	0.26
**Number of positive nodes**															
None	Ref			Ref			Ref			Ref			Ref		
1-3	1.08	0.55, 2.1	0.82	1.47	0.82, 2.62	0.20	0.97	0.67, 1.41	0.89	1.15	0.52, 2.56	0.73	0.99	0.70, 1.42	0.97
≥4	1.52	0.41, 5.7	0.53	0.62	0.20, 1.91	0.40	1.47	0.67, 3.23	0.34	0.48	0.11, 2.20	0.35	1.29	0.60, 2.77	0.51
**Hormone-receptor positivity**															
ER- and PR -	Ref			Ref			Ref			Ref			Ref		
ER + and/or PR+	1.14	0.63, 2.06	0.66	1.19	0.70, 2.01	0.52	1.26	0.91, 1.73	0.17	1.25	0.60, 2.59	0.55	1.38	1.01, 1.88	0.04
**HER2/Neu status**															
Negative	Ref			Ref			Ref			Ref			Ref		
Positive	1.11	0.57, 2.16	0.76	1.12	0.67, 1.88	0.66	0.69	0.50, 0.97	0.03	0.62	0.27, 1.42	0.26	1.05	0.76, 1.43	0.78
Not performed	0.96	0.29, 2.93	0.89	1.56	0.69, 3.54	0.29	1.93	1.0, 3.71	0.05	1.73	0.65, 4.60	0.27	2.11	1.21, 3.69	0.009
**Surgery type**															
Conserving or none	Ref			Ref			Ref			Ref			Ref		
Mastectomy	1.12	0.67, 1.89	0.67	0.86	0.56, 1.32	0.48	1.09	0.81, 1.46	0.57	1.03	0.56, 1.88	0.93	1.22	0.93, 1.60	0.15
**Treatment status**															
None	Ref			Ref			Ref			Ref			Ref		
Chemotherapy only	1.08	0.53, 2.20	0.84	0.55	0.31, 0.96	0.03	0.89	0.62, 1.28	0.53	0.45	0.21, 0.96	0.04	0.71	0.50, 1.0	0.05
Radiation only	1.22	0.61, 2.41	0.57	0.72	0.43, 1.21	0.22	1.14	0.72, 1.51	0.83	0.87	0.43, 1.75	0.69	1.02	0.73, 1.43	0.90
Both	0.94	0.42, 2.10	0.88	0.67	0.35, 1.29	0.23	0.89	0.57, 1.39	0.60	0.46	0.18, 1.18	0.10	0.75	0.50, 1.15	0.19
**BMI**															
<25 kg/m^2^	Ref			Ref			Ref			Ref			Ref		
25-30 kg/m^2^	0.68	0.42, 1.09	0.11	0.98	0.67, 1.45	0.93	0.81	0.63, 1.05	0.10	1.21	0.72, 2.05	0.47	0.86	0.67, 1.09	0.20
>30 kg/m^2^	1.04	0.63, 1.72	0.88	1.39	0.92, 2.09	0.12	0.81	0.61, 1.07	0.14	1.53	0.88, 2.67	0.13	0.96	0.74, 1.24	0.74
**Fruit and vegetable intake**															
<35 servings/week	Ref			Ref			Ref			Ref			Ref		
≥35 servings/week	1.14	0.76, 1.72	0.52	1.42	1.02, 1.97	0.04	1.60	1.28, 1.99	<.0001	0.91	0.58, 1.42	0.68	1.48	1.20, 1.83	.0002
**Hrs/wk of moderate-vigorous activity**															
<2.5	Ref			Ref			Ref			Ref			Ref		
2.5 - 5	0.89	0.52, 1.52	0.56	1.05	0.68, 1.64	0.82	1.42	1.06, 1.93	0.02	1.05	0.54, 2.07	0.88	1.27	0.96, 1.69	0.10
>5	0.89	0.55, 1.43	0.74	0.94	0.64, 1.39	0.77	1.18	0.91, 1.54	0.27	1.69	0.98, 2.91	0.06	1.17	0.91, 1.50	0.23
**Smoking status**															
Never	Ref			Ref			Ref			Ref			Ref		
Ever	1.19	0.79, 1.78	0.40	1.40	1.0, 1.95	0.05	1.40	1.12, 1.74	0.003	1.61	1.03, 2.52	0.04	1.0	0.81, 1.23	0.96

^a^Among Pathways participants with both baseline and 6 month follow up questionnaire and who were users prior to breast cancer diagnosis.

^b^Beta Carotene not included in the table because insufficient number of users prior to diagnosis (n = 20).

^c^Odds ratios and 95% confidence intervals are based on multivariable logistic regression models adjusted for all covariates.

**Table 4:** Predictors of discontinuing vitamin/mineral supplement use by the 6-month follow up

**Covariates**	**Multivitamins (n = 1,563)**			**Vitamin C (n = 723)**			**Calcium (n = 542)**			**Vitamin E (n = 496)**			**Vitamin D (n = 287)**		
	**OR**	**95% CI**	**P-value**	**OR**	**95% CI**	**P-value**	**OR**	**95% CI**	**P-value**	**OR**	**95% CI**	**P-value**	**OR**	**95% CI**	**P-value**
**Age**															
<50 years	Ref			Ref			Ref			Ref			Ref		
50-59 years	0.73	0.52, 1.04	0.08	0.81	0.48, 1.38	0.44	0.51	0.28, 0.93	0.03	0.87	0.39, 1.92	0.72	0.69	0.22, 2.13	0.52
60-69 years	0.58	0.40, 0.85	0.005	0.98	0.58, 1.67	0.94	0.59	0.32, 1.08	0.09	0.73	0.33, 1.60	0.43	0.45	0.14, 1.42	0.17
70+ years	0.37	0.23, 0.56	<.0001	1.26	0.69, 2.30	0.46	0.73	0.36, 1.46	0.37	0.77	0.32, 1.85	0.56	0.86	0.25, 2.92	0.81
**Race**															
Non-Hispanic white	Ref			Ref			Ref			Ref			Ref		
African American	1.38	0.82, 2.31	0.22	1.83	0.86, 3.89	0.12	1.77	0.75, 4.16	0.19	1.93	0.72, 5.19	0.19	0.70	0.18, 2.70	0.61
Asian	0.86	0.56, 1.31	0.48	1.71	0.92, 3.16	0.09	1.71	0.87, 3.37	0.12	1.48	0.60, 3.63	0.40	1.58	0.51, 4.89	0.43
Hispanic	1.18	0.76, 1.84	0.45	0.99	0.54, 1.82	0.97	0.88	0.43, 1.80	0.73	0.80	0.37, 1.74	0.57	3.07	1.19, 7.94	0.02
**Education**															
High School or less	Ref			Ref			Ref			Ref			Ref		
Some college	0.60	0.40, 0.90	0.01	1.47	0.84, 2.55	0.18	1.32	0.66, 2.63	0.44	1.33	0.68, 2.61	0.41	2.17	0.67, 6.99	0.20
College graduate	0.56	0.37, 0.87	0.01	1.17	0.65, 2.10	0.61	0.57	0.27, 1.18	0.13	0.68	0.33, 1.39	0.29	1.05	0.31, 3.58	0.94
Post graduate	0.60	0.39, 0.94	0.03	1.29	0.71, 2.33	0.40	1.10	0.54, 2.26	0.79	0.91	0.44, 1.89	0.80	0.73	0.21, 2.50	0.61
**Household income**															
≤$25,000	Ref			Ref			Ref			Ref			Ref		
$25,000-49,999	1.21	0.72, 2.04	0.47	1.20	0.63, 2.31	0.58	0.46	0.20, 1.05	0.06	1.60	0.76, 3.36	0.21	1.80	0.56, 5.75	0.32
$50,000-89,999	0.88	0.53, 1.47	0.63	1.38	0.72, 2.63	0.33	0.50	0.22, 1.11	0.09	2.39	1.15, 4.95	0.02	0.88	0.28, 2.78	0.82
$90,000+	0.94	0.56, 1.60	0.83	2.43	1.24, 4.78	0.01	0.74	0.32, 1.68	0.46	2.91	1.35, 6.25	0.01	0.81	0.25, 2.67	0.73
**Family history of breast cancer**															
No	Ref			Ref			Ref			Ref			Ref		
Yes	0.93	0.69, 1.27	0.65	0.88	0.60, 1.30	0.51	0.67	0.41, 1.08	0.10	1.18	0.71, 1.94	0.53	0.82	0.38, 1.77	0.61
**Stage at diagnosis**															
I	Ref			Ref			Ref			Ref			Ref		
II	1.05	0.72, 1.52	0.81	0.89	0.55, 1.44	0.64	0.69	0.37, 1.29	0.24	0.82	0.42, 1.60	0.56	1.01	0.37, 2.79	0.98
III+	1.69	0.75, 3.83	0.21	1.77	0.49, 6.38	0.39	0.33	0.07, 1.52	0.16	0.45	0.08, 2.53	0.36	1.97	0.21, 18.57	0.55
**Number of positive nodes**															
None	Ref			Ref			Ref			Ref			Ref		
1-3	1.12	0.73, 1.71	0.60	1.17	0.66, 2.07	0.59	1.35	0.67, 2.73	0.40	1.27	0.58, 2.79	0.56	1.50	0.45, 5.00	0.51
≥4	1.09	0.47, 2.56	0.84	1.05	0.27, 4.03	0.94	4.47	0.95, 21.18	0.06	1.49	0.24, 9.17	0.67	0.93	0.08, 10.23	0.95
**Hormone-receptor positivity**															
ER- and PR -	Ref			Ref			Ref			Ref			Ref		
ER + and/or PR+	0.80	0.56, 1.14	0.21	0.76	0.48, 1.21	0.25	0.74	0.43, 1.29	0.29	1.30	0.69, 2.45	0.42	0.68	0.28, 1.62	0.38
**HER2/Neu status**															
Negative	Ref			Ref			Ref			Ref			Ref		
Positive	1.04	0.73, 1.49	0.81	1.31	0.78, 2.20	0.31	0.61	0.33, 1.13	0.12	0.79	0.40, 1.58	0.50	0.92	0.32, 2.63	0.88
Not performed	1.46	0.76, 2.80	0.26	0.80	0.33, 1.97	0.63	0.90	0.32, 2.54	0.84	0.87	0.24, 3.12	0.83	0.82	0.14, 4.89	0.83
**Surgery type**															
Conserving or none	Ref			Ref			Ref			Ref			Ref		
Mastectomy	0.94	0.68, 1.29	0.69	0.59	0.38, 0.90	0.01	0.71	0.43, 1.17	0.18	0.58	0.33, 1.03	0.06	0.66	0.28, 1.54	0.34
**Treatment status**															
None	Ref			Ref			Ref			Ref			Ref		
Chemotherapy only	1.57	1.02, 2.43	0.04	1.71	0.99, 2.97	0.05	1.48	0.74, 2.95	0.26	2.29	1.09, 4.79	0.03	0.97	0.31, 3.08	0.96
Radiation only	1.25	0.81, 1.93	0.32	0.85	0.51, 1.41	0.52	0.76	0.41, 1.42	0.39	0.51	0.27, 0.98	0.04	0.54	0.19, 1.56	0.25
Both	1.05	0.61, 1.81	0.85	1.31	0.67, 2.58	0.43	1.20	0.55, 2.65	0.65	0.99	0.42, 2.32	0.98	2.77	0.92, 8.39	0.07
**BMI**															
<25 kg/m^2^	Ref			Ref			Ref			Ref			Ref		
25-30 kg/m^2^	1.03	0.77, 1.39	0.83	0.78	0.53, 1.14	0.20	1.01	0.62, 1.64	0.97	1.23	0.74, 2.02	0.43	0.65	0.29, 1.46	0.29
>30 kg/m^2^	0.93	0.67, 1.29	0.68	0.78	0.51, 1.20	0.26	1.53	0.93, 2.52	0.10	1.01	0.57, 1.79	0.97	1.22	0.53, 2.80	0.64
**Fruit and vegetable intake**															
<35 servings/week	Ref			Ref			Ref			Ref			Ref		
≥35 servings/week	0.85	0.66, 1.11	0.23	1.19	0.85, 1.66	0.31	0.70	0.47, 1.04	0.08	0.82	0.54, 1.25	0.36	0.48	0.25, 0.94	0.03
**Hrs/wk of moderate- vigorous activity**															
<2.5	Ref			Ref			Ref			Ref			Ref		
2.5 - 5	0.71	0.49, 1.04	0.08	1.00	0.62, 1.60	0.99	1.63	0.93, 2.87	0.09	0.80	0.44, 1.46	0.46	1.92	0.77, 4.79	0.16
>5	1.15	0.85, 1.57	0.37	0.82	0.54, 1.24	0.34	1.62	0.99, 2.65	0.06	0.74	0.42, 1.28	0.27	1.05	0.48, 2.27	0.91
**Smoking status**															
Never	Ref			Ref			Ref			Ref			Ref		
Ever	0.95	0.73, 1.23	0.68	0.81	0.58, 1.12	0.20	0.88	0.59, 1.30	0.51	0.70	0.46, 1.08	0.10	0.96	0.49, 1.88	0.90

^a^Among Pathways participants with both baseline and 6 month follow up questionnaire and who were non-users prior to breast cancer diagnosis.

^b^Beta Carotene not included in the table because insufficient number of users prior to diagnosis (n = 20).

^c^Odds ratios and 95% confidence intervals are based on multivariable logistic regression models adjusted for all covariates.

**Table 5:** Predictors of continuous vitamin/mineral supplement use by the 6-month follow up

**Covariates**	**Multivitamins (n = 1,563)**			**Vitamin C (n = 723)**			**Calcium (n = 542)**			**Vitamin E (n = 496)**			**Vitamin D (n = 287)**		
	**OR**	**95% CI**	**P-value**	**OR**	**95% CI**	**P-value**	**OR**	**95% CI**	**P-value**	**OR**	**95% CI**	**P-value**	**OR**	**95% CI**	**P-value**
**Age**															
<50 years	Ref			Ref			Ref			Ref			Ref		
50-59 years	1.28	0.91, 1.8	0.16	1.26	0.84, 1.89	0.27	2.06	1.34, 3.16	0.001	1.78	0.91, 3.48	0.09	2.99	1.65, 5.4	.0003
60-69 years	1.10	0.77, 1.56	0.61	1.27	0.84, 1.92	0.27	2.48	1.6, 3.86	<.0001	2.54	1.32, 4.89	0.01	5.31	2.95, 9.54	<.0001
70+ years	1.51	0.99, 2.31	0.05	1.28	0.8, 2.04	0.30	2.36	1.41, 3.96	0.001	2.35	1.15, 4.79	0.02	7.14	3.75, 13.61	<.0001
**Race**															
Non-Hispanic white	Ref			Ref			Ref			Ref			Ref		
African American	0.66	0.4, 1.08	0.10	0.58	0.32, 1.05	0.07	0.59	0.31, 1.15	0.12	0.57	0.24, 1.4	0.22	2.34	1.3, 4.2	0.005
Asian	0.79	0.54, 1.14	0.21	0.57	0.35, 0.91	0.02	0.61	0.37, 1	0.05	0.47	0.23, 0.98	0.04	0.80	0.46, 1.38	0.42
Hispanic	0.54	0.37, 0.78	0.001	0.73	0.47, 1.12	0.15	0.66	0.42, 1.06	0.09	0.87	0.48, 1.57	0.64	0.99	0.56, 1.75	0.97
**Education**															
High School or less	Ref			Ref			Ref			Ref			Ref		
Some college	1.82	1.27, 2.61	0.001	0.88	0.6, 1.31	0.53	0.97	0.6, 1.55	0.89	0.96	0.56, 1.66	0.89	1.02	0.59, 1.76	0.95
College graduate	1.63	1.11, 2.39	0.01	1.01	0.66, 1.54	0.98	1.38	0.84, 2.25	0.20	1.28	0.72, 2.26	0.40	1.48	0.84, 2.62	0.17
Post graduate	1.90	1.27, 2.85	0.002	1.12	0.73, 1.73	0.60	1.42	0.85, 2.37	0.18	1.12	0.62, 2.02	0.72	2.53	1.44, 4.46	0.001
**Household income**															
≤$25,000	Ref			Ref			Ref			Ref			Ref		
$25,000-49,999	1.03	0.65, 1.63	0.91	1.38	0.87, 2.17	0.17	2.15	1.17, 3.94	0.01	0.75	0.43, 1.29	0.30	1.08	0.61, 1.9	0.79
$50,000-89,999	1.26	0.81, 1.97	0.30	1.19	0.76, 1.87	0.45	2.13	1.17, 3.88	0.01	0.56	0.33, 0.98	0.04	1.29	0.75, 2.22	0.36
$90,000+	0.99	0.62, 1.57	0.97	0.74	0.45, 1.2	0.22	2.05	1.1, 3.8	0.02	0.49	0.27, 0.88	0.02	1.36	0.77, 2.41	0.28
**Family history of breast cancer**															
No	Ref			Ref			Ref			Ref			Ref		
Yes	1.05	0.8, 1.39	0.71	1.05	0.78, 1.4	0.77	1.04	0.75, 1.45	0.82	0.86	0.57, 1.3	0.48	1.28	0.91, 1.8	0.17
**Stage at diagnosis**															
I	Ref			Ref			Ref			Ref			Ref		
II	1.11	0.8, 1.56	0.53	1.00	0.69, 1.43	0.98	0.72	0.47, 1.09	0.12	0.97	0.58, 1.62	0.91	0.81	0.52, 1.28	0.37
III+	2.00	0.81, 4.96	0.14	0.64	0.22, 1.86	0.41	0.74	0.27, 2	0.55	1.58	0.45, 5.53	0.47	0.70	0.23, 2.13	0.53
**Number of positive nodes**															
None	Ref			Ref			Ref			Ref			Ref		
1-3	1.02	0.69, 1.52	0.91	0.88	0.57, 1.37	0.57	0.98	0.6, 1.58	0.92	1.10	0.59, 2.05	0.78	1.27	0.74, 2.18	0.38
≥4	0.51	0.2, 1.29	0.15	0.86	0.29, 2.53	0.78	1.09	0.38, 3.11	0.87	0.90	0.25, 3.31	0.87	1.27	0.39, 4.07	0.69
**Hormone-receptor positivity**															
ER- and PR -	Ref			Ref			Ref			Ref			Ref		
ER + and/or PR+	1.15	0.83, 1.61	0.41	0.97	0.66, 1.43	0.88	1.22	0.8, 1.85	0.36	0.84	0.5, 1.4	0.49	1.31	0.82, 2.11	0.26
**HER2/Neu status**															
Negative	Ref			Ref			Ref			Ref			Ref		
Positive	1.52	1.05, 2.2	0.03	0.83	0.54, 1.26	0.38	0.99	0.65, 1.5	0.96	1.01	0.58, 1.76	0.97	0.77	0.45, 1.32	0.34
Not performed	0.86	0.45, 1.63	0.64	1.37	0.71, 2.66	0.35	1.90	0.84, 4.32	0.13	0.89	0.34, 2.32	0.81	1.70	0.7, 4.11	0.24
**Surgery type**															
Conserving or none	Ref			Ref			Ref			Ref			Ref		
Mastectomy	0.96	0.71, 1.3	0.80	1.28	0.9, 1.82	0.17	0.84	0.58, 1.23	0.37	1.22	0.75, 2	0.43	1.43	0.94, 2.18	0.10
**Treatment status**															
None	Ref			Ref			Ref			Ref			Ref		
Chemotherapy only	0.74	0.5, 1.1	0.13	0.71	0.47, 1.09	0.11	0.95	0.58, 1.54	0.83	0.55	0.29, 1.02	0.06	0.57	0.34, 0.96	0.03
Radiation only	0.99	0.67, 1.46	0.96	1.05	0.69, 1.58	0.83	0.89	0.56, 1.44	0.65	1.35	0.77, 2.35	0.29	0.93	0.57, 1.52	0.78
Both	0.58	0.36, 0.91	0.02	0.64	0.37, 1.12	0.12	0.66	0.36, 1.21	0.18	0.84	0.4, 1.74	0.63	0.84	0.44, 1.58	0.58
**BMI**															
<25 kg/m^2^	Ref			Ref			Ref			Ref			Ref		
25-30 kg/m^2^	0.85	0.65, 1.12	0.25	0.90	0.67, 1.21	0.50	0.92	0.66, 1.28	0.61	0.90	0.61, 1.34	0.61	1.10	0.77, 1.55	0.61
>30 kg/m^2^	1.12	0.83, 1.52	0.47	0.93	0.68, 1.29	0.67	0.88	0.61, 1.26	0.48	0.86	0.56, 1.33	0.49	0.82	0.55, 1.23	0.33
**Fruit and vegetable intake**															
<35 servings/week	Ref			Ref			Ref			Ref			Ref		
≥35 servings/week	1.59	1.26, 2.02	.0001	1.06	0.82, 1.36	0.68	1.45	1.09, 1.93	0.01	1.11	0.78, 1.56	0.57	1.59	1.17, 2.16	0.003
**Hrs/wk of moderate-vigorous activity**															
<2.5	Ref			Ref			Ref			Ref			Ref		
2.5 - 5	1.15	0.84, 1.57	0.38	1.08	0.76, 1.55	0.66	1.01	0.68, 1.51	0.94	1.77	1.09, 2.87	0.02	0.74	0.47, 1.16	0.19
>5	1.20	0.91, 1.59	0.20	1.18	0.87, 1.61	0.29	1.04	0.74, 1.46	0.83	1.44	0.93, 2.23	0.11	1.07	0.74, 1.54	0.72
**Smoking status**															
Never	Ref			Ref			Ref			Ref			Ref		
Ever	0.94	0.74, 1.19	0.58	1.27	0.98, 1.63	0.07	1.18	0.89, 1.58	0.26	1.25	0.89, 1.75	0.20	0.89	0.66, 1.2	0.43

^a^Among Pathways participants with both baseline and 6 month follow up questionnaire.

^b^ Comparing continuous users to non-users prior to breast cancer diagnosis.

^c^Beta Carotene not included in the table because insufficient number of users prior to diagnosis (n = 20).

^d^Odds ratios and 95% confidence intervals are based on multivariable logistic regression models adjusted for all covariates.

#### Demographics

Women over age 50 years were more likely to initiate vitamin D and calcium; continue to use calcium, vitamin E, and vitamin D; and less likely to initiate vitamin E or discontinue multivitamin supplements. Race was not strongly associated with supplement use. Higher education predicted both initiating and not discontinuing multivitamins. Higher household income was associated with not initiating multivitamins, discontinuing vitamin E, and continuously using multivitamins and vitamin D.

#### Clinical characteristics

Family history of breast cancer and several clinical characteristics, including stage, number of positive nodes, and hormone receptor status, were not associated with supplement initiation, although there was a trend against initiating multivitamins among those with higher stage tumors.

There were few consistencies observed across treatment groups. Examining supplement use by treatments received, individuals undergoing chemotherapy were less likely to initiate the antioxidants vitamin C and vitamin E, continue use of vitamin D, and more likely to discontinue multivitamins. Individuals undergoing both chemotherapy and radiation were less likely to continue using multivitamins. There were differences in supplement use between women who had different surgical procedures. Among women who had a mastectomy, there was a trend toward continuing rather than discontinuing most supplements.

#### Lifestyle factors

Neither BMI nor physical activity was consistently associated with initiating or discontinuing supplement use. Women consuming higher amounts of fruits/vegetables were more likely to initiate vitamin C, vitamin D, and calcium, but not vitamin E or multivitamins.

## Discussion

In a large prospective cohort study of women in Northern California diagnosed with first primary breast cancer, we observed high use and initiation of vitamin and mineral supplements in the six months following diagnosis. The most commonly used supplements were multivitamins, calcium, vitamin C, and vitamin D. Only a small percent of women discontinued using specific supplements during this time. On average, the doses used by women far exceeded the recommended intake levels by the IOM. In our models, women who initiated supplements were generally highly educated, consumed more fruits and vegetables, were more likely to have ever smoked cigarettes, and were less likely to have received chemotherapy; whereas women who discontinued were more likely to be under age 50, less likely to have higher education, and did not undergo a complete mastectomy. Continuous users were more likely be older, have higher education, and consume more fruits and vegetables. In general, supplement use was greater among women who consumed more fruits and vegetables, suggesting that supplement use is higher in the population that needs supplementation the least.

Several prior studies have reported similarly high rates of vitamin/mineral supplementation among breast cancer patients and survivors [1,3,4,7,9-12,19,25-28]. Studies that examined use of specific supplements describe similar prevalence of multivitamin use, but much higher use of vitamins C and E than we observed [1,4,26]. Since supplement use data were collected over ten years ago for these studies, differences may represent new recommendations regarding antioxidant use during chemotherapy [29]. One study reported higher rates of overall supplement use among breast cancer survivors compared to population-based controls [1].

In general, we report that women in the study population were somewhat more likely to discontinue than initiate supplement use. Since the commonly discontinued supplements in this population were multivitamins and vitamins C and E, we hypothesize that this difference is explained by our prospective data collection among women while they are receiving treatment. Accordingly, a recent report of high-risk breast cancer patients participating in a clinical trial observed comparable changes in use during treatment, namely decreased use of multivitamins, vitamin C, and vitamin E, consistent use of folate, and increases in vitamins B6 and B12 [19]. However, they did not observe similar increases in vitamin D and calcium use, likely explained by younger participant age. The findings that different measures of socioeconomic status (i.e., education and income) are associated with opposite levels of use and non-use of some of the supplements is perplexing. We think that a main take home message from these findings is that different constellations of factors are associated with use of different supplements and that it is important to consider this when interpreting dietary supplement data.

The evidence-base for use of supplements during cancer treatment is inconsistent. Women are likely increasing their use of calcium and vitamin D for bone health. Prior studies suggest that calcium and vitamin D supplementation are safe for breast cancer patients [14,30]. In the Pathways Study population, African American women reported higher use of iron and vitamin B12 before diagnosis compared to other women, likely reflecting elevated anemia risk found in African Americans [31]. While a single study has shown that B12 may reduce breast cancer risk in premenopausal women [32], and other studies have shown that iron may promote carcinogenesis [33,34], enough data do not exist to reach a clear consensus to infer causation about the risks or benefits of these dietary supplements. Folic acid and B12 are necessary to regulate DNA methylation and prevent DNA damage, but supplementation during cancer treatment is controversial [35]. Chemotherapeutic agents such as methotrexate and 5-fluorouracil disrupt folate channels in order to promote tumor apoptosis, and supplementation during cancer treatment has the potential to reduce cytotoxicity [36].

Data on the effects of antioxidant use during treatment, such as vitamin E, vitamin C, and carotenoids, are mixed. There is concern that antioxidants during chemotherapy and radiation therapy may reduce treatment effectiveness [29]. One hypothesis is that antioxidant supplements may effectively block the otherwise effective pro-oxidant therapies and therefore reduce treatment effects [37]. However, their effects on cancer outcomes remain unclear. Observational studies report inconsistent results on use of antioxidants and risk of recurrence and mortality in breast cancer patients [4,6]. Use of carotenoid supplements is of concern because of large human trials showing increased risk of lung cancer in those assigned to take beta-carotene [38,39]. Use of beta-carotene as an individual supplement was not common in this population, with less than two percent reporting use after diagnosis, but many women likely use multivitamins containing beta-carotene. A recent cohort study of breast cancer patients showed increased risk of mortality among those taking combination carotenoids [4] but not multivitamins [40]. Future studies utilizing information of the timing and dosage of supplement use during treatment will help clarify whether supplement interacts with or modifies the effect of conventional cancer treatments.

This study presents methodological improvements over previous studies. While several prior studies were conducted within large established cohorts, this study specifically examined changes in behavior after diagnosis, distinguishing between supplement initiators and discontinuers from continuous and never users. Therefore, it was possible to examine changes in behavior, which may affect survival outcomes and be especially relevant in establishing evidence-based recommendations for patients. This study is one of the first to prospectively collect data on supplement use shortly after diagnosis, including time during treatment. This approach differs from many prior studies where patients were queried on their supplement use far after diagnosis. Several previous studies were also limited by crude measurements of supplement intake, which make it difficult to detect small associations with cancer outcomes that are likely confounded by lifestyle and socioeconomic factors. Here we report detailed information on dose, allowing for comprehensive assessment of use patterns among continuous users, initiators, and discontinuers.

Strengths of this study include the large population of recently diagnosed women, an abundance of information on supplement use and relevant covariates, and prospective data collection. However, there are limitations. First, the participation rate in the Pathways study was 47% of the invited sample. Thus, we cannot rule out the possibility that women who enrolled in the study were healthier and more health conscious than those who did not enroll, potentially biasing our results away from the null if participants are more likely to use dietary supplements than non-participants. Differences by age and BMI at breast cancer diagnosis were minimal when comparing the enrolled to unenrolled women, respectively: average age at diagnosis 59.6 y (range: 23.6-94.8 y) vs. 61.6 y (range: 21.0-99.8 y) and 34% obese (BMI ≥ 30 kg/m^2^) vs. 32%. Further, the enrolled women appear largely representative of the overall breast cancer population, with a slight shift to earlier stage disease. Unenrolled women were more likely to be African American or Asian, compared to enrolled women. Second, not all women completed the 6 month follow-up questionnaire. Compared to those who completed the 6 month follow-up questionnaire, non-completers were younger, more likely to self-report as African American, Asian or Hispanic, had less education, and were diagnosed with higher stage disease. As with the non-enrollers, if the non-completers were less health conscious than the completers, study results on supplement use could be biased away from the null. In addition, this study was restricted to a single geographic region with the median education level being college graduate, which may limit generalizability of results.

A strength of the study is the detailed assessment of supplement use. Trained interviewers assessed supplement use at baseline, whereas follow-up questionnaires were completed by participants. A prior study reported low validity of self-report for some supplements, compared with the gold standard of interviewer inspection of container labels [41]. However, most of the error was derived from extracting single micronutrient compositions from multivitamin supplements and distinguishing multiple vitamins and single supplements. A subsequent study reported high validity and reproducibility of self-administered mailed questionnaires, compared with in-person interview, and higher self-reported intakes were linearly correlated with increasing blood concentrations [42]. Prevalence of supplement use prior to diagnosis may be overestimated because it was assessed after diagnosis, and may not accurately represent usual intake before diagnosis.

## Conclusions

While previous studies have shown that breast cancer patients are very likely to use vitamin and mineral supplements, we present the first large-scale study with detailed information on changes in supplement use following diagnosis in a multi-ethnic population. Current guidelines for cancer survivors recommend achieving a healthy weight; consuming a diet high in fruits, vegetables and whole grains while limiting processed foods and those high in saturated fat; participating in at least 150 minutes of physical activity per week; and not taking supplements to prevent recurrence [43]. A better understanding of predictors of supplement initiation and discontinuation is beneficial as we continue to examine the effects of supplement use on breast cancer outcomes. These dietary supplements have the potential to modify the effects of treatment on outcomes, including breast cancer recurrence and survival. Therefore, future studies need to test possible interactions between supplements and treatment in order to inform guidelines on the use of dietary supplements during breast cancer treatment.

## Competing interests

The authors declare that they have no competing interests.

## Authors’ contributions

HG designed the presented study and contributed to writing all sections of the text. MLK contributed to the design and implementation of the original Pathways study as well as analysis and writing of this manuscript. IJE conducted analysis and contributed to the writing of the manuscript. GS contributed to the writing of the manuscript. JMR contributed to the study implementation as well as the analysis and writing of the manuscript. ATW conducted analysis for this manuscript. ML, KJS, CBA, DLH, and AIN contributed to the study design. LHK contributed to the study design, implementation, analysis and writing of the manuscript. All authors have read and approved the submission of this manuscript. This manuscript has not been published and is not under consideration for publication elsewhere.

## Pre-publication history

The pre-publication history for this paper can be accessed here:

http://www.biomedcentral.com/1471-2407/14/382/prepub
